# Association between chronic bronchitis and increased breast cancer risk in female patients

**DOI:** 10.5588/pha.26.0013

**Published:** 2026-08-24

**Authors:** X. Ruan, D. Chen, S. Cai, H. Yang, B. Wang

**Affiliations:** Department of Clinical Laboratory, The Second Affiliated Hospital of Soochow University, Suzhou, Jiangsu, China.

**Keywords:** NHANES, pulmonary function, inflammatory markers, airway inflammation

## Abstract

**BACKGROUND:**

Respiratory diseases and breast cancer both pose significant threats to women’s health, but the association between them remains unclear.

**METHODS:**

This analysis utilised data including 25,132 adult female participants. After adjusting variables, a logistic regression model assessed the association between respiratory diseases and breast cancer risk. We investigated the nonlinear dose–response relationship between pulmonary function tests and breast cancer, and analysed the association of pulmonary function test with breast cancer risk across different racial groups. We explored the heterogeneous characteristics of key metabolic and inflammatory markers in different groups.

**RESULTS:**

In the fully adjusted model, chronic bronchitis was associated with prevalent breast cancer. Restricted cubic spline curve revealed elevated breast cancer risk when pulmonary function test met the following thresholds: FEF25–75% < 2,405 ml/s, FEV1/FVC < 0.794, and FEV3/FVC < 0.926, with stronger associations between pulmonary function and breast cancer risk found among non-Hispanic whites and blacks. Serum lipid metabolism and inflammation markers showed significant elevation.

**CONCLUSION:**

Chronic bronchitis exhibits a potential correlation with breast cancer risk. In non-Hispanic whites and non-Hispanic blacks, declining pulmonary function may associate with increased breast cancer risk.

Breast cancer is the most common malignancy in women worldwide. According to the American Cancer Society’s 2024 report, the risk of breast cancer among American women rose at an average annual rate of 1% between 2012 and 2021.^1^ Chronic inflammation and metabolic dysfunction are recognised as causal factors in breast cancer pathogenesis. Some studies indicate lipid metabolism abnormalities are closely associated with an increased risk of breast cancer.^2^ In addition, chronic inflammation creates an inflammatory microenvironment via pro-inflammatory cytokines and reactive oxygen species (ROS), which promote proliferation of epithelial cells and impair DNA damage repair.^3^ Respiratory diseases, such as chronic bronchitis, emphysema, and asthma are characterised by chronic airway inflammation,^4^ which leads to impaired pulmonary function.^5^ Inflammation serves as a risk factor for breast cancer and represents a characteristic pathological alteration in respiratory diseases.^6^ In addition, some patients with respiratory diseases experience activity limitations and reduced exercise capacity due to declining pulmonary function,^7^ and dietary imbalances in these patients may contribute to metabolic abnormalities.

However, direct evidence regarding the association between chronic bronchitis and breast cancer remains limited at present. Existing studies have rarely examined racial heterogeneity in the association between pulmonary function and prevalent breast cancer. The primary objective of this study was to examine the association between respiratory disease and breast cancer risk among adult women and then assess potential nonlinear associations between pulmonary function measures and breast cancer, racial heterogeneity in these associations, and differences in metabolic and inflammatory markers across disease status groups.

## METHODS

Data for this study were obtained from NHANES. Conducted by the National Center for Health Statistics (NCHS) under the U.S. Centers for Disease Control and Prevention, NHANES is a nationally representative survey.^8^ Data are collected through standardised household interviews, detailed physical examinations, and laboratory tests.

### Study population

**FIGURE 1.**
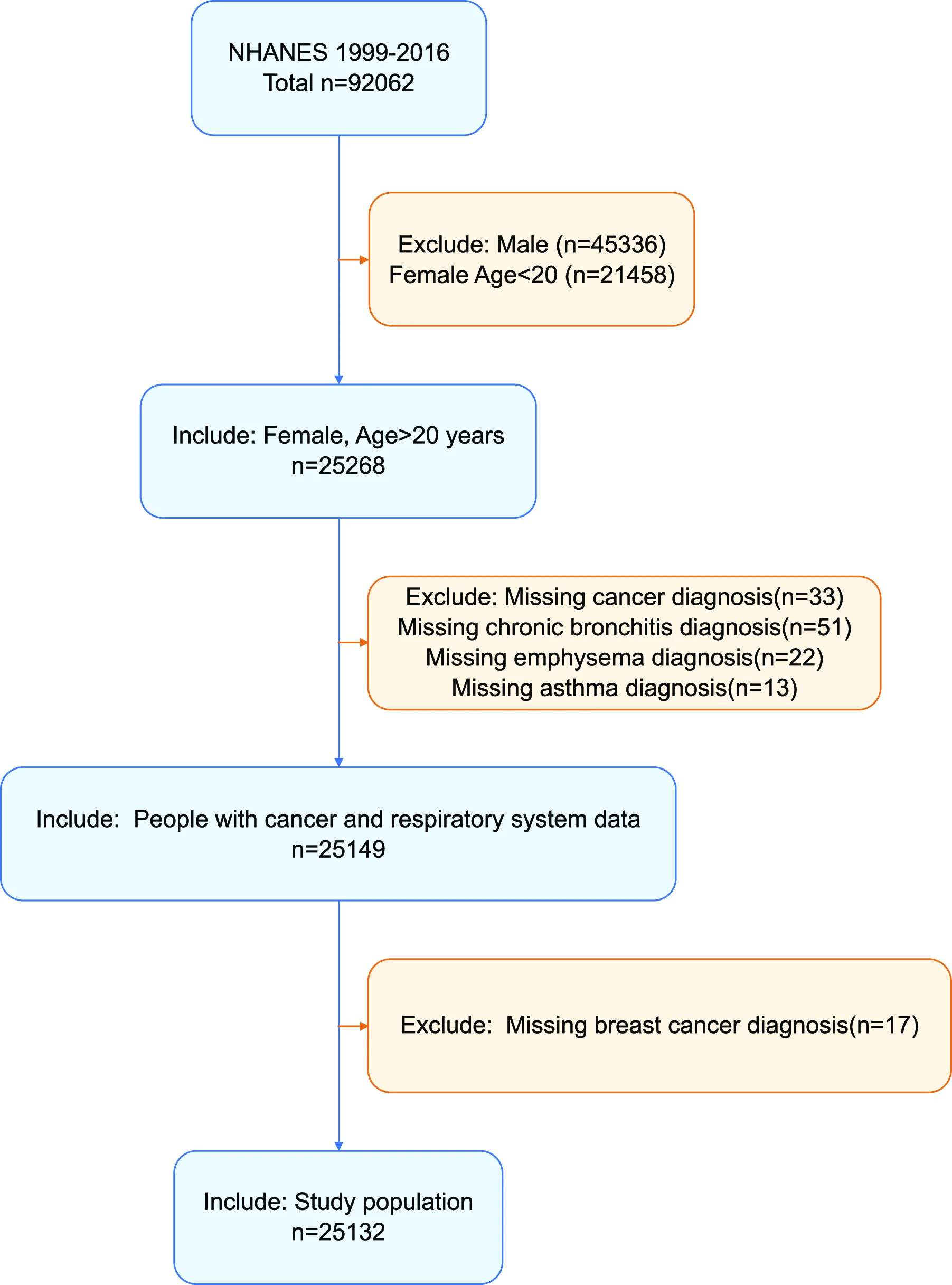
Flow chart of selection process for the study population in NHANES from 1999 to 2016.

The data were derived from NHANES conducted from 1999 to 2016. First, from 92,062 total participants, people under 20 years of age were excluded, yielding 25,268 preliminarily eligible adult women. Subsequently, participants lacking cancer, chronic bronchitis, emphysema, or asthma diagnosis information were further excluded. Finally, those without breast cancer diagnosis information were removed. 25,132 female participants were ultimately included in the study population (Figure 1).

### Diagnostic criteria for respiratory system diseases and breast cancer in NHANES

In NHANES, participants were asked, ‘Has a doctor ever told you that you have cancer?’ Those answering ‘yes’ were further asked about the specific type of cancer. This study defined breast cancer patients as those explicitly self-reporting ‘breast cancer’.^9^ The diagnoses of chronic bronchitis and asthma were based on participants’ responses. Emphysema diagnosis used composite criteria, primarily based on chest X-ray interpretation results.

### Covariates

Variable selection was based on known epidemiological associations with respiratory diseases and breast cancer.^9^ Age was divided into three groups: 20–40 years, 41–65 years, and >65 years. Educational attainment was categorised into three levels: Lesshigh, high, and abovehigh. Poverty–income ratio (PIR) was categorised as low income, moderate income, and high income. Marital status was categorised as married, divorced, or never married. Race was categorised as Mexican American, other Hispanic, non-Hispanic white, non-Hispanic black, and other race. Body mass index (BMI) was categorised according to WHO standards: thin, normal weight, overweight, and obesity. Smoking status was categorised as never smoker (lifetime consumption <100 cigarettes), previous smoker, or now smoker.

### Statistical analysis

All analyses accounted for the complex survey design of NHANES by incorporating the appropriate sample weights, strata, and primary sampling units. Continuous variables were expressed as medians with interquartile range (IQR), with comparisons using the Wilcoxon rank-sum test. Categorical variables were described by weighted frequency, with intergroup differences assessed using the χ² test. Missing covariate data were handled by generating five imputed datasets, and the final estimates were pooled using Rubin’s rules. Logistic regression models analysed associations between respiratory disease and breast cancer risk. Results are presented as odds ratio (OR) with 95% confidence interval (CI). Restricted cubic spline (RCS) analyses were performed to assess potential nonlinear relationships. When *P* value was <0.05, the result was considered statistically significant. Participants were categorised into four groups based on respiratory disease and breast cancer status. Differences in serum total cholesterol (TC), triglycerides (TG), ferritin (Fer), and systemic inflammatory response index (SIRI) were compared across groups, with boxplots illustrating medians and IQRs. All statistical analyses were performed using R statistical software (version 4.5.1; http://www.R-project.org).

### Ethical statement

The study protocol is approved by the NCHS Research Ethics Review Committee.^10,11^

## RESULTS

The median age in breast cancer patients was significantly higher than those in the non-breast cancer participants. The proportion of non-Hispanic whites in the breast cancer patients was significantly higher than that in the non-breast cancer participant. Breast cancer patients exhibited a significantly higher proportion of divorced, previous smoking history, chronic bronchitis prevalence, and emphysema prevalence compared to the non-breast cancer participants. Laboratory test indicators showed that breast cancer patients had higher levels of TC, TG, Fer, and SIRI compared to non-breast cancer participants (Table 1).

**TABLE 1.** Weighted baseline characteristics of participants.

Characteristic	The general population, n = 25,132	Non-breast cancer participants, n = 24,410	Breast cancer patients, n = 722	*P* value
Continuous age (year)	47 (34, 65)	46 (34, 60)	67 (57, 77)	<0.001
Categorical age (year)				<0.001
20–40	9,112 (36%)	9,096 (37%)	16 (2.2%)	
41–65	10,026 (40%)	9,768 (40%)	258 (36%)	
65–85	5,994 (24%)	5,546 (23%)	448 (62%)	
Education level				0.2
Lesshigh	6,909 (27%)	6,733 (28%)	176 (24%)	
High	5,638 (22%)	5,465 (22%)	173 (24%)	
Abovehigh	12,585 (50%)	12,212 (50%)	373 (52%)	
PIR				<0.001
≤1.3	8,354 (33%)	8,159 (33%)	195 (27%)	
1.3–3.5	9,484 (38%)	9,204 (38%)	280 (39%)	
≥3.5	7,294 (29%)	7,047 (29%)	247 (34%)	
Marital status				<0.001
Married	13,846 (55%)	13,505 (55%)	341 (47%)	
Divorced	7,432 (30%)	7,094 (29%)	338 (47%)	
Unmarried	3,854 (15%)	3,811 (16%)	43 (6.0%)	
Race				<0.001
Mexican American	4,477 (18%)	4,409 (18%)	68 (9.4%)	
Other Hispanic	2,167 (8.6%)	2,120 (8.7%)	47 (6.5%)	
Non-Hispanic White	11,254 (45%)	10,798 (44%)	456 (63%)	
Non-Hispanic Black	5,181 (21%)	5,064 (21%)	117 (16%)	
Other Race	2,053 (8.2%)	2,019 (8.3%)	34 (4.7%)	
BMI (kg/m^2^)	27.40 (23.44, 32.73)	27.40 (23.43,32.80)	27.40 (23.77, 31.79)	0.7
Smoking status				<0.001
Never	15,894 (63%)	15,488 (64%)	406 (56%)	
Previous	4,858 (19%)	4,620 (19%)	238 (33%)	
Now	4,347 (17%)	4,270 (18%)	77 (11%)	
Chronic bronchitis				<0.001
Yes	1,835 (7.3%)	1,752 (7.2%)	83 (11%)	
No	23,297 (93%)	22,658 (93%)	639 (89%)	
Emphysema status				<0.001
Yes	424 (1.7%)	396 (1.6%)	28 (3.9%)	
No	24,708 (98%)	24,014 (98%)	694 (96%)	
Asthma status				0.8
Yes	3,703 (15%)	3,594 (15%)	109 (15%)	
No	21,429 (85%)	20,816 (85%)	613 (85%)	
Laboratory tests				
TC (mmol/L)	5.07 (4.40, 5.79)	5.07 (4.40, 5.79)	5.20 (4.55, 6.03)	0.013
TG (mmol/L)	1.15 (0.81, 1.70)	1.15 (0.80, 1.69)	1.36 (0.98, 1.91)	<0.001
Fer (μg/L)	43.00 (23.00,78.00)	42.70 (23.00,78.00)	57.00 (34.00,133.00)	0.003
SIRI	1.00 (0.70, 1.46)	1.00 (0.70, 1.45)	1.19 (0.82, 1.88)	<0.001

*P* < 0.05 indicates statistically significant differences.

PIR = poverty–income ratio; BMI = body mass index; TC = total cholesterol; TG = triglycerides; Fer = ferritin; SIRI = systemic inflammatory response index.

### Association between respiratory disease and breast cancer

Increasing age showed a positive association with breast cancer risk. PIR ≤ 1.3 was associated with lower odds of breast cancer. Unmarried women exhibited a higher odd of breast cancer. Non-Hispanic whites had higher odds than other racial groups. Chronic bronchitis and emphysema may be associated with prevalent breast cancer, but asthma showed no significant association.

### Association between chronic bronchitis and breast cancer

**FIGURE 2.**
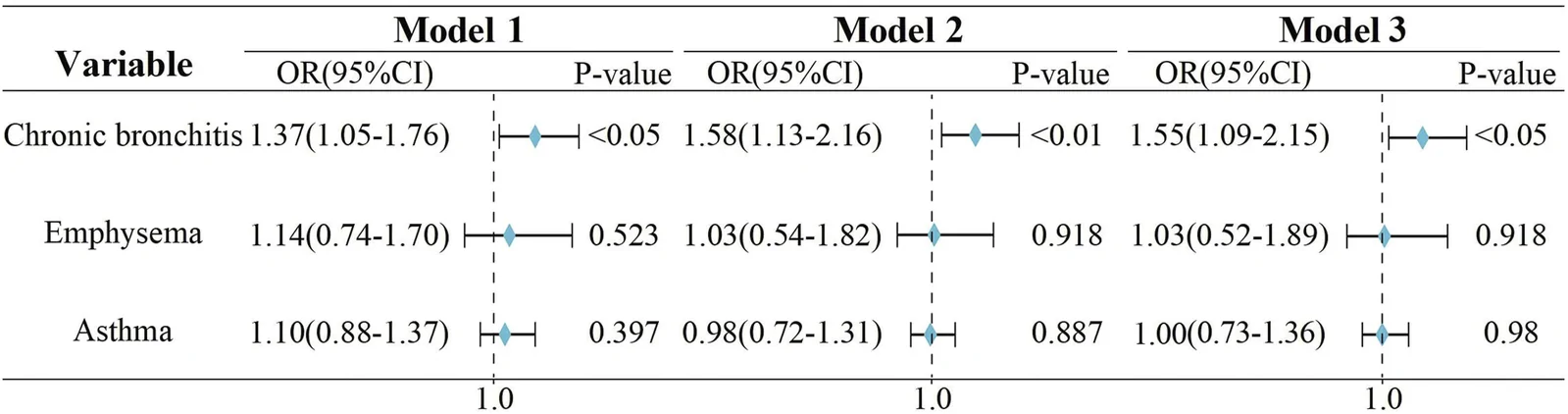
Multivariate logistic regression model between respiratory diseases and breast cancer. *P* <0.05 indicates statistically significant differences. OR = odds ratio; CI = confidence interval.

To examine the stability of these associations, adjusted logistic regression models were constructed, as shown in Figure 2: Model 1 only adjusted for age; Model 2 adjusted for age, race, education level, PIR, and marital status; Model 3 further adjusted for BMI and smoking status. Model 3 showed that chronic bronchitis was associated with prevalent breast cancer (OR = 1.55, 95% CI: 1.09–2.15), *P* < 0.05. The association between emphysema and prevalent breast cancer in Model 3 was not statistically significant, suggesting that the univariate association may have been influenced by confounding.

### Analysis of the influence of pulmonary function thresholds on breast cancer

**FIGURE 3.**
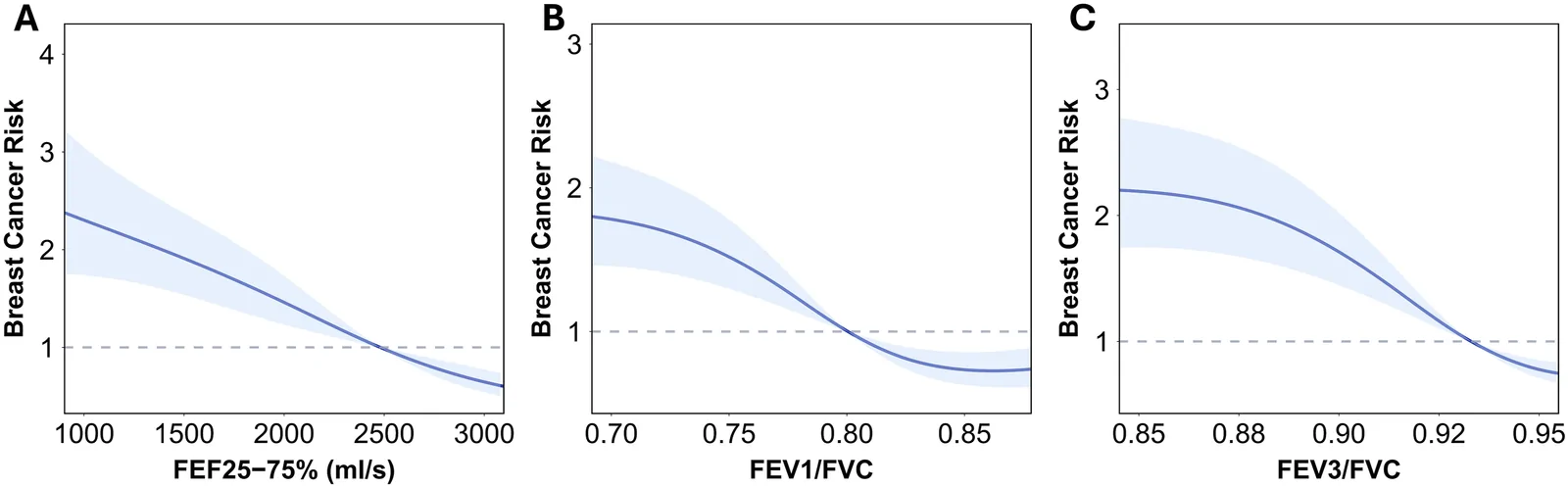
Association between pulmonary function and breast cancer risk. **A)** RCS of FEF25–75% and breast cancer risk; **B)** RCS of FEV1/FVC and breast cancer risk; **C)** RCS of FEV3/FVC and breast cancer risk.

Pulmonary function tests were only assessed during the 2007–2012 survey period. The cohort was divided into three groups: the general population (n = 8,898), the non-breast cancer participants (n = 8,627), and the breast cancer patients (n = 271). We used RCS to identify thresholds for three pulmonary function indicators. Threshold effects were observed between prevalent breast cancer and three pulmonary function measures: FEF25–75% (2,405 ml/s, OR = 1.797, 95% CI: 1.440–2.258), FEV1/FVC (0.794, OR = 1.470, 95% CI: 1.177–1.846), and FEV3/FVC (0.926, OR = 1.812, 95% CI: 1.419–2.328). Below these thresholds, prevalent breast cancer increased (Figure 3A–C).

### Threshold effect analysis of pulmonary function across different racial groups

**FIGURE 4.**
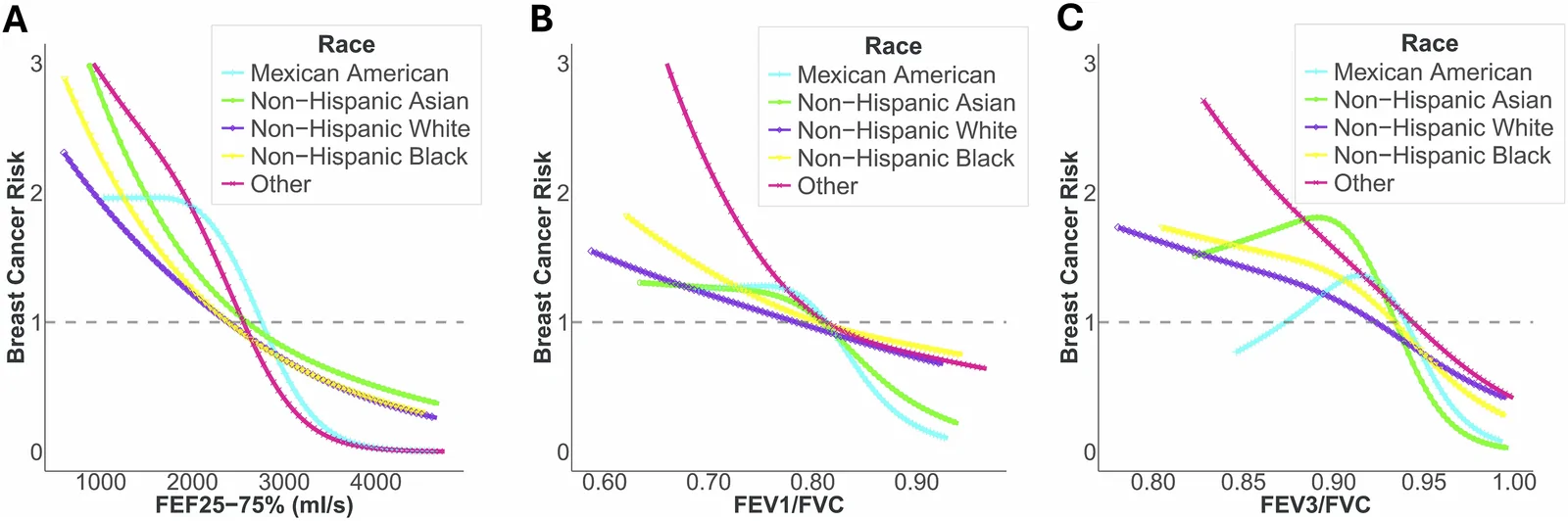
Association between pulmonary function and breast cancer risk across racial groups. **A)** RCS of FEF25–75% and breast cancer risk across racial groups; **B)** RCS of FEV1/FVC and breast cancer risk across racial groups; **C)** RCS of FEV3/FVC and breast cancer risk across racial groups.

In non-Hispanic white and non-Hispanic black, significant associations with prevalent breast cancer were observed for FEF25–75%, FEV1/FVC, and FEV3/FVC, *P* < 0.05. Mexican Americans and non-Hispanic showed no significant association with prevalent breast cancer. In other racial groups, FEV1/FVC showed no significant association. In conclusion, the association between pulmonary function indicators and breast cancer risk differs among racial groups (Figure 4A–C).

### Laboratory tests among different disease groups

Fer and SIRI were higher in the BC and CB groups relative to Neither group. The Both group exhibited the highest Fer and SIRI level. Lipid metabolism disorders and increased inflammatory responses are often exhibited in patients with both chronic bronchitis and breast cancer.

## DISCUSSION

We found a potential correlation between chronic bronchitis and breast cancer risk. Decreased pulmonary function was associated with elevated breast cancer risk. Clinically, decreased FEF25–75% reflects small airway dysfunction, and lower FEV1/FVC and FEV3/FVC indicate greater airflow limitation.^12^ Reduced pulmonary function reflects greater systemic hypoxia, and chronic inflammatory burden; these implicated in tumour-promoting microenvironments.^13^

An additional finding was the heterogeneity of these associations across racial groups. Stronger associations between reduced pulmonary function and prevalent breast cancer were observed among non-Hispanic White and non-Hispanic Black women, and the associations were not significant in other racial groups. These differences may stem from other underlying factors; for example, gene polymorphisms may influence pulmonary development, and imbalances in health care resources may also be relevant across racial groups.^14,15^ Both group exhibited the most pronounced metabolic and inflammatory disturbances. This dyslipidaemia may enhance cell membrane fluidity, promote steroid synthesis, and activate inflammatory pathways.^16^ Elevated Fer promotes ROS production via iron-catalysed Fenton reactions, and SIRI independently predicts survival in women with breast cancer.^17,18^

This study provides a more comprehensive assessment by integrating respiratory disease status, pulmonary function, racial heterogeneity, and metabolic-inflammatory profiles in an analytical framework.^19^ However, several limitations should be considered. First, the cross-sectional design precludes any inference regarding temporality between chronic bronchitis, lower pulmonary function, and breast cancer. Second, both breast cancer and chronic bronchitis were primarily identified based on self-reported data; participants may not accurately report diagnoses, which may have introduced recall bias. Third, although multiple covariates were adjusted for, residual confounding cannot be excluded. Future research should focus on conducting prospective cohort studies to clarify the effects of chronic bronchitis and pulmonary function on breast cancer incidence and performing external validation across diverse populations.^20^

## CONCLUSION

Chronic bronchitis and reduced pulmonary function were associated with prevalent breast cancer in adult women. These findings highlight a potential association between respiratory health and breast cancer, suggesting that respiratory status deserves greater attention.
